# Verfügbarkeit und Zielsetzungen digitalisierungsbezogener Lehrkräftefortbildungen für naturwissenschaftliche Lehrkräfte in Deutschland

**DOI:** 10.1007/s40573-021-00134-1

**Published:** 2021-10-05

**Authors:** Charlotte Diepolder, Holger Weitzel, Johannes Huwer, Sarah Lukas

**Affiliations:** 1 Pädagogische Hochschule Weingarten, Kirchplatz 2, 88250 Weingarten, Deutschland; 2 Universität Konstanz, 78457 Konstanz, Deutschland

**Keywords:** Lehrerprofessionalisierung, Digitalisierung, DiKoLAN, Schulschließungen, Distanzunterricht, Digitalisierungsbezogene Qualifikation, Teacher professionalisation, Digitalisation, DiKoLAN, School closure, Distance learning, Digitalisation related qualification

## Abstract

Lehrkräfte sind essentiell für die Integration digitaler Technologien in Schule und Unterricht. Ihre Qualifizierung für diese Aufgabe ist daher in allen Phasen der Lehrkräfteausbildung von zentraler Bedeutung, zumal nur etwa ein Drittel der derzeit im aktiven Schuldienst befindlichen Lehrkräfte während des Studiums Lerngelegenheiten zu einem digitalisierungsbezogenen Kompetenzaufbau hatte. Trotz des Wunsches nach einer digitalisierungsbezogenen Qualifikation besuchen deutsche Lehrkräfte jedoch vergleichsweise selten Fortbildungen. Neben fehlender Passung des Fortbildungsangebots könnte für diese Diskrepanz ein unzureichendes Angebot verantwortlich sein. Wir untersuchen in dieser Studie, welche Fortbildungsangebote in 12 von 16 Bundesländern explizit für naturwissenschaftliche Lehrkräfte über staatliche Fortbildungskataloge angeboten wurden, welche Kompetenzbereiche des Orientierungsrahmens „Digitale Kompetenzen für das Lehramt in den Naturwissenschaften“ (DiKoLAN) die Fortbildungen adressieren und inwieweit die in den Fortbildungen adressierten didaktischen Funktionen die Lehrkräfte bei der Ausbringung von Distanzunterricht während der Schulschließungen 2020 hätten unterstützen können. Insgesamt werden 90 Fortbildungen identifiziert und damit zu wenige, um die Zielsetzungen der Kultusminister Konferenz-Strategie „Bildung in der digitalen Welt“ angemessen zu verfolgen. Aus den Angeboten lassen sich kaum Hinweise auf Faktoren ablesen, die der individuellen Lehrkraft einen kumulativen Kompetenzaufbau ermöglichen. Fast alle Fortbildungsangebote adressieren für Naturwissenschaftslehrkräfte relevante Kompetenzbereiche des DiKoLAN-Orientierungsrahmens, ein Teil ist geeignet, Lehrkräfte die didaktischen Funktionen zu vermitteln, die auch bei der Ausbringung von Distanzunterricht genutzt werden können.

## Abstract (en)

Teachers are essential for the integration of digital technologies into schools and lessons. Their qualification for this task is therefore of central importance in all phases of teacher training, especially since only about one third of teachers currently in active teaching service had learning opportunities for digitization-related competence building during their studies. Despite the desire for a digitization-related qualification, however, German teachers attend continuing education courses comparatively rarely. In addition to a lack of fit in the training offered, an insufficient offer could be responsible for this discrepancy. In this study, we investigate which in-service training courses were explicitly offered to science teachers via state in-service training catalogues in 12 of 16 German states, which competence areas of the orientation framework “Digitale Kompetenzen für das Lehramt in den Naturwissenschaften (DiKoLAN)” the in-service trainings address, and to what extent the didactic functions addressed in the in-service trainings could have supported teachers in providing distance learning during the 2020 school closures. A total of 90 in-service trainings are identified, too few to adequately address the objectives of the KMK strategy “Bildung in der digitalen Welt/Education in the Digital World”. The offers show little evidence of factors that enable individual teachers to build cumulative competence. Almost all of the in-service training offers address competence areas of the DiKoLAN orientation framework that are relevant for science teachers, and some of them are suitable for teaching teachers the didactic functions that can also be used in distance learning.

## Ausgangslage und Relevanz der Forschungsfrage

Gleich zweimal, ab Mitte März 2020 und dann wieder ab Mitte Dezember 2020, mussten alle Schulen in Deutschland ihre Türen für den Präsenzunterricht schließen. Die Lehrkräfte wurden dadurch vor die Herausforderung gestellt, ihren Unterricht binnen kürzester Zeit auf alternative und hier insbesondere digitale Formate umzustellen.

Die Schulschließung traf in Deutschland auf ein Schulsystem, dessen technische Ausstattung (z. B. schulische Geräteausstattung und Geräteverfügbarkeit für Lehrkräfte, WLAN-Verfügbarkeit, Verfügbarkeit von Lernmanagementsystemen, IT-Support) unter dem Durchschnitt von Ländern mit vergleichbarem Entwicklungsstand lag (Eickelmann et al. 2019a; GEW 2020). Gleiches galt für die Nutzungshäufigkeit digitaler Medien im Unterricht, in denen sich Deutschland im Ländervergleich am unteren Ende des Rankings wiederfand (Eickelmann et al. 2019a, S. 17). Begleitend zur Schulschließung im Frühjahr entstanden eine Reihe von Studien zur Beschreibung des Umgangs der Schulen, Lehrkräfte, Schüler*innen und Eltern mit den plötzlich auftretenden digitalen Herausforderungen (vgl. Fickermann und Edelstein 2021). Deren Ergebnisse sind vor dem Hintergrund der methodischen Herausforderungen solcher Studien zu bewerten (Huber und Helm 2020). Der Ende April 2020 erschienene Schulbarometer berichtete, dass mehr als die Hälfte der Schulleitungen (56 %) die technische Ausstattung ihrer Schule als nicht ausreichend für digitalen Unterricht betrachtete (Huber et al. 2020). Die befragten Lehrkräfte fühlten sich für digitale Unterrichtsformate nur bedingt vorbereitet (ebd., S. 52), jedoch wurde die erzwungene Konfrontation mit digitalen Technologien in Zeiten der Schulschließungen als wichtiger Schritt zur eigenen Professionalisierung gewertet (ebd., S. 53). Die Kommunikation mit Schüler*innen und Eltern erfolgte am häufigsten über E‑Mail (67,7 %). Lernplattformen und Software für synchronen virtuellen Unterricht wurden am häufigsten von Schulen genutzt, in denen ein Abitur abgelegt werden kann und eher dann, wenn die Schulen bereits im Vorfeld der Schulschließungen auf eine bessere technische Infrastruktur zugreifen konnten und häufiger digitale Medien für den Unterricht eingesetzt hatten (Lorenz et al. 2020).

In einer Befragung unter Thüringer Lehrkräften aller Schulstufen (Dreer und Kracke 2020) ergab sich ein ähnliches Bild, jedoch dominierten hier noch stärker E‑Mail (89 %) und Telefon (56,0 %) als Kommunikationsmittel. Cloud-Dienste (18,8 %) und Lernplattformen (14,8 %) als am häufigsten genutzte andere digitale Dienste wurden dagegen seltener als in der Studie von Lorenz et al. (2020) genannt. Die Thüringer Lehrkräfte gaben an, während der Schulschließungen bevorzugt die digitalen Technologien zu nutzen, mit denen sie bereits vor dem Lockdown Erfahrungen sammeln konnten. Ihre Vorerfahrungen zum Umgang mit Lernplattformen und fachspezifischen Lernangeboten schätzten sie als eher gering ein.

Analog zu Ergebnissen der ICILS-Studien wurden digitale Technologien auch während des Distanzunterrichts selten für formatives Assessment und Feedback eingesetzt (Eickelmann et al. 2019a; König et al. 2020). Zu ähnlichen Ergebnissen kommt eine qualitative Studie mit 68 befragten Lehrkräften aus drei Bundesländern: Es werden bekannte Technologien und Unterrichtspraktiken verwendet, das Potenzial digitaler Medien, Unterricht interaktiv zu gestalten und gezieltes Feedback zu geben, wird seltener genutzt und als aufwändiger empfunden (Eichhorn et al. 2020).

In Schulen, die bereits im Vorfeld der Pandemie stärker digitalisiert waren, zeigten sich die Lehrkräfte durch die Umgestaltung der Lerninhalte auf rein digitale Formate weniger belastet, die Lernangebote erreichten die Schüler*innen besser und die Lehrkräfte konnten auch keine Vergrößerung der Bildungsschere beobachten (Eickelmann et al. 2020).

### Einsatz digitaler Medien im Distanzunterricht während der Schulschließungen

Studien zur Situation des naturwissenschaftlichen Unterrichts während der weltweiten Schulschließungen als Folge der Covid-19 Pandemie sind rar. Eine in Estland durchgeführte qualitative Studie mit fünf Lehrkräften berichtet Ergebnisse zur Nutzung digitaler Technologien und zur Initiierung kollaborativen Arbeitens (Rannastu-Avalos und Siiman 2020). Alle befragten Lehrkräfte verwendeten Videokonferenz-Programme und Lernmanagementplattformen, empfanden beide Werkzeuge jedoch als wenig hilfreich zur Förderung kollaborativer Zusammenarbeit zwischen den Schüler*innen. Auf Basis einer Fallanalyse mit einer einzelnen Lehrkraft beschreibt eine amerikanische Studie Herausforderungen für die Lehrkraft, ihre Schüler*innen zu motivieren und geeignetes individuelles Feedback zu geben. Fehlende Möglichkeiten zum Experimentieren während des Distanzunterrichts versuchte die Lehrkraft durch häusliche Experimente („backyard science explorations“) zu kompensieren (Kaden 2020). In einer weiteren Studie wurde der Wissenszuwachs zu chemischen Reaktionen im Distanzunterricht gemessen. Mit Arbeitsblättern, die über Augmented Reality Elemente angereichert waren, konnte ein größerer Wissenszuwachs erzielt werden als bei der Arbeit mit analogen Arbeitsblättern (Probst et al. 2020).

Während der Schulschließungen beschäftigten sich Schüler*innen häufiger mit den Hauptfächern (Mathematik, Deutsch, Fremdsprachen) als mit den Naturwissenschaften (Accelerom AG 2020). Die für Biologie, Chemie und Physik aufgewendete Arbeitszeit hing ihrerseits von der Schulform ab und war am höchsten an Gymnasien. Unter den Fächern konnte ein Gefälle zwischen Biologie und den beiden anderen Naturwissenschaften festgestellt werden (Biologie: Gymnasium [Gym] > 60 %, Realschule [RS] > 45 % und < 50 %, Hauptschule [HS] > 30 %; Chemie: Gym > 35 % und < 40 %, RS > 20 % und < 30 %, HS > 20 %; Physik: Gym > 45 % und < 50 %, RS ca. 30 %, HS ca. 20 % [Accelerom AG 2020, S. 16]).

### Digitalisierungsbezogene Kompetenzen und Lerngelegenheiten

Damit im naturwissenschaftlichen Unterricht digitale und digital unterstütze Lehr-Lernszenarien realisiert werden können (vgl. Abschnitt *Einsatz digitaler Medien im Distanzunterricht während der Schulschließungen*), müssen Lehrkräfte entsprechende Kompetenzen besitzen. Lehrkräfte gelten als „keystone species“ für die Integration digitaler Technologien in schulisches Lernen (Davis et al. 2013, S. 439). Ihre darauf bezogene Qualifizierung ist eine zentrale Aufgabe der Lehrerbildung (van Ackeren et al. 2019), die in Deutschland erstmals für alle Bundesländer mit der Strategie „Bildung in der digitalen Welt“ (Kultusminister Konferenz [KMK] 2016) adressiert wurde. Der KMK-Strategie folgend streben die Länder an, zukünftige Lehrkräfte bereits während der universitären Ausbildung zur adäquaten Nutzung digitaler Technologien zu qualifizieren. Für Lehrkräfte im Schuldienst, die während ihrer Ausbildung keine digitalisierungsbezogenen Lerngelegenheiten hatten, sollen Fortbildungsmöglichkeiten bereitgestellt werden, die deren Kompetenzaufbau absichern und darüber hinaus geeignet sein sollen, bereits vorhandene Kompetenzen zu erweitern und zu vertiefen (KMK 2016, S. 30). Grundlage zur Erreichung der Zielsetzungen ist die Entwicklung und Implementation geeigneter Kompetenzrahmen. Wie groß die daraus resultierenden qualitativen wie quantitativen Herausforderungen für die staatliche Lehrerfortbildung sind, zeigt unter anderem die Tatsache, dass unter den derzeit in Deutschland unterrichtenden Lehrkräften lediglich ein Viertel während der ersten und zweiten Lehrerbildungsphase digitalisierungsbezogene Lerngelegenheiten hatte. Damit liegt der Anteil deutscher Lehrkräfte deutlich unter dem Mittelwert (47,5 %) der an der ICILS-Studie teilnehmenden Länder (Eickelmann et al. 2019b).

Trotz fehlender Möglichkeiten zum Kompetenzaufbau während der ersten Lehrerbildungsphasen besuchte laut der ICILS-Studie von 2018 nur etwa ein Drittel der Lehrkräfte digitalisierungsbezogene Fortbildungen. Ihr Anteil ist damit niedriger als in vergleichbaren Ländern (Gerick et al. 2019). Nach einer aktuellen Studie unter GEW-Mitgliedern aus dem Zeitraum kurz vor dem Lockdown begründen Lehrkräfte ihre Nicht-Teilnahme an Fortbildungsangeboten am häufigsten mit dem Fehlen relevanter Angebote (49 %) oder fehlenden zeitlichen Ressourcen (41 %) (GEW 2020, S. 41). Die Studie liefert damit Hinweise, dass der ausgeprägte Wunsch vieler Lehrkräfte nach digitalisierungsbezogenen Fortbildungen (vgl. Hoffmann und Richter 2016; Eickelmann et al. 2016) mit dem verfügbaren Angebot nicht zufriedenstellend abgedeckt ist.

Wie bedeutsam die Wahrnehmung von Fortbildungen für die Qualifikation von Lehrkräften ist, zeigt eine vertiefte Analyse der Daten von ICILS 2018 zu so genannten digitalen Optimalschulen (Eickelmann und Drossel 2020). Damit sind nicht-gymnasiale Schulen gemeint, die entgegen der sonstigen Schulformabhängigkeit ihren Schüler*innen besonders erfolgreich digitale Kompetenzen vermitteln. An diesen Schulen nehmen Lehrkräfte häufiger an Fortbildungen insbesondere zum fächerspezifischen Einsatz digitaler Medien teil (48 % im Vergleich zu 31 % an allen Schulen in Deutschland).

### Digitalisierungsbezogene Kompetenzbereiche für Lehrkräfte

Die digitalisierungsbezogene Professionalisierung von Lehrkräften ist ein langfristig zu betrachtender Unterrichts- und Schulentwicklungsprozess, bei dem individuelle Faktoren der Lehrkräfte wie Wissen, Einstellungen und Selbstwirksamkeitsüberzeugungen mit externen Stimuli wie Fortbildungen und Möglichkeiten zur Anwendung neu erlernten Wissens in Einklang gebracht und hinsichtlich der Wirksamkeit auf Lernprozesse von Schüler*innen reflektiert werden müssen (Petko et al. 2018). Eickelmann und Gerick (2020, S. 155) identifizieren vor diesem Hintergrund vier Zielperspektiven schulischen Lernens mit digitalen Medien:

1. Vermittlung von Fertigkeiten im Umgang mit digitalen Medien
2. Nutzung digitaler Medien zur Verbesserung fachlichen Lernens
3. Entwicklung und Umsetzung neuer Formen des Unterrichtens mit digitalen Medien
4. Förderung des Medienkompetenzerwerbs.

Die Zielperspektiven 2 und 3 beziehen sich auf die Nutzung digitaler Medien in fachlichen Lehr-Lernprozessen. Sie umfassen Kompetenzen, um „digitale Medien in ihrem jeweiligen Fachunterricht professionell und didaktisch sinnvoll [zu] nutzen“ (Kultusminister Konferenz 2016, S. 25). Zur Beschreibung dieser Kompetenzen greifen verschiedene Bundesländer auf unterschiedliche Rahmenmodelle (z. B. DigCompEdu, Redecker 2017; TPACK, Koehler und Mishra 2009) zurück. Das TPACK-Modell adressiert im Wesentlichen die oben beschriebenen Zielperspektiven 2 und 3. Das Akronym TPACK steht für *Technological Pedagogical and Content Knowledge* und definiert aufbauend auf Shulmans (1986) Beschreibung von PCK die drei Wissensbereiche Content Knowledge (CK), Pedagogical Knowledge (PK) und Technological Knowledge (TK) als wesentlich für professionell handelnde Lehrkräfte. Die Wissensbereiche überschneiden sich teilweise, sodass daraus letztlich sieben Teilbereiche entstehen. Dies sind neben CK, PK und TK die Bereiche Pedagogical Content Knowledge (PCK), Technological Content Knowledge (TCK), Technological Pedagogical Knowledge (TPK) und aus der Überschneidung aller Bereiche folgend das Technological Pedagogical Content Knowledge (TPACK). TPACK beschreibt die Kompetenz, technologisches, pädagogisches und fachliches Wissen adäquat zur Planung, Durchführung und Reflexion technologiebezogenen domänenspezifischen Unterrichts nutzen zu können. Da TPACK zunächst nicht auf ein bestimmtes Fach oder eine Fächergruppe bezogen ist, müssen die Kompetenzbeschreibungen fachbezogen aufgeschlüsselt werden.

### Digitale Kompetenzen von Lehramtsstudierenden der Naturwissenschaften (DiKoLAN)

Eine solche Ausdifferenzierung des TPACK-Modells für die „digitalen Kompetenzen von Lehramtsstudierenden der Naturwissenschaften“ stellt der DiKoLAN-Orientierungsrahmen dar, der von der Arbeitsgruppe „Digitale Basiskompetenzen“ der Joachim Herz Stiftung entwickelt wurde (Becker et al. 2020, S. 16). Durch den Bezug auf die naturwissenschaftlichen Fächer wird eine Spezifizierung der Kompetenzanforderungen erreicht. Zudem ermöglicht er eine Organisation des Kompetenzaufbaus über die drei Phasen der Lehrerbildung hinweg. Der Orientierungsrahmen soll aber zunächst durch „klar formulierte Kompetenzbereiche, -niveaus und -ziele, […] [die] Erstellung von Curricula für die universitäre Phase der Lehrerbildung [ermöglichen]. Darüber hinaus ergeben sich weitere Anwendungsfelder und Nutzungspotenziale. Im Vergleich zum Ziel der Bildungsstandards (Messung individuell vorhandener Kompetenzen am normativen Rahmen der Standards) ermöglichen die im Orientierungsrahmen DiKoLAN formulierten Kompetenzen eine Evaluierung von Kompetenzständen und -entwicklungsprozessen als wesentliches Element der Professionalisierung bei Lehramtsstudierenden.“ (Becker et al. 2020, S. 26).

Da ein Großteil der Lehrkräfte im aktiven Dienst keine digitalisierungsbezogenen Lerngelegenheiten während der universitären Ausbildung hatte, kann DiKoLAN auch für diese Gruppe eine wichtige Orientierungshilfe darstellen, um die naturwissenschaftsspezifischen Kompetenzbereiche TPACK, TPK, TCK und TK klar zu identifizieren und darauf aufbauend ein Weiter- und Fortbildungsangebot zu gestalten.

DiKoLAN besteht aus sieben Kompetenzbereichen, welche durch rechtliche Rahmenbedingungen und technische Basiskompetenzen eingeklammert sind:

1. Dokumentation
2. Präsentation
3. Kommunikation/Kollaboration
4. Recherche/Bewertung
5. Messwert‑/Datenerfassung
6. Datenverarbeitung
7. Simulation/Modellierung

Jeder dieser Kompetenzbereiche ist wiederum untergliedert in die Bereiche TPACK, TPK, TCK und TK. Diese vier Bereiche beinhalten die Kompetenzniveaus „Nennen“ „Beschreiben“ (inkl. notwendigen Vorgehen) sowie „Anwenden/Durchführen“ (praktische und funktionale Realisierung), anhand derer sich kompetentes Handeln zeigt. Insbesondere die Bereiche TPACK, TPK, TCK sind dabei in vorwiegend fachdidaktischen Veranstaltungen zu finden, da die unterrichtliche Praxis im Fach eine entscheidende Rolle spielt (Petko et al. 2018).

### Ziel der Studie

Zusammenfassend kann ein großer Bedarf an Fortbildungen zur Schaffung und Vertiefung digitalisierungsbezogener Kompetenzen bei Lehrkräften konstatiert werden, die Voraussetzung ist für den flexiblen und fachspezifischen Einsatz digitaler Medien im Präsenz- wie im Distanzunterricht. Für die Bereitstellung des Angebots sind die Bundesländer verantwortlich. Wir untersuchten in dieser Studie, welche staatlichen Fortbildungsangebote für Lehrkräfte naturwissenschaftlicher Fächer im Vorfeld des Lockdowns verfügbar waren, um sich für die Herausforderungen des Einsatzes digitaler Medien im Fachunterricht zu qualifizieren, die schließlich auch unter den Bedingungen des Distanzunterrichts essentiell sein können. Zur domänenspezifischen Einordnung fragen wir darüber hinaus, welche für naturwissenschaftlichen Unterricht bedeutsamen Kompetenzbereiche mit den Fortbildungsangeboten adressiert werden.

## Methode

Grundlage der Analyse sind die in staatlichen Fortbildungskatalogen für die Sekundarstufe I und II (ohne Berufsschulen) aufgeführten digitalisierungsbezogenen Fortbildungen in den drei Naturwissenschaften aus 12 der 16 Bundesländer. Die Auswahl erfolgte mit dem Ziel, solche Fortbildungen zu identifizieren, die für alle naturwissenschaftlichen Lehrkräfte eines Bundeslandes digital sichtbar und damit prinzipiell zugänglich sind. Nicht aufgenommen wurden die Angebote freier Anbieter zum einen, weil deren Sichtbarkeit für alle Schulen über die Hierarchiekette aus Kultusministerium, Regierungspräsidium, Schulamt, Schulleitung bis hin zu den Lehrkräften nicht sichergestellt werden kann und es dem Zufall überlassen ist, ob eine Lehrkraft beispielsweise über einen Flyer im Lehrerzimmer oder über eine E‑Mail von der Schulleitung von den Angeboten erfährt. Zum anderen ist Lehrkräftefortbildung eine Aufgabe der Kultusministerien, die darüber entscheiden, welche Angebote sie in ihren Fortbildungskatalog aufnehmen. Ferner sind die Kultusministerien über den Digitalpakt dazu angehalten, digitalisierungsbezogene Angebote zur Förderung von TPACK der Lehrkräfte anzubieten. Der von uns verwendete Katalog ist damit das Referenzangebot, auf das sich Lehrkräfte beziehen können.

Wir haben uns aus drei Gründen dafür entschieden, die Datenaufnahme auf das Schuljahr direkt vor der Corona Pandemie zu begrenzen, die einerseits retrospektiv wie prospektiv begründet sind:

1. Im Schuljahr 2019/2020 trat der Digitalpakt offiziell in Kraft und gab den Digitalisierungsbemühungen der Länder neue Verbindlichkeit, die sich auch in einer größeren Anzahl an Fortbildungsangeboten hätte spiegeln können.
2. Die Entwicklung von digitalen Werkzeugen (wie Programmen zur AR-Gestaltung) ist hochdynamisch. Länger zurückliegende Fortbildungen können sich auf digitale Werkzeuge beziehen, die zum Zeitpunkt der Schulschließung bereits nicht mehr verfügbar waren.
3. Zum Teil entstand erst während des Lockdowns der Bedarf an Werkzeugen (wie z. B. Videokonferenzlösungen), die im Präsenzunterricht zuvor irrelevant waren.

Die Gesamtzahl der digitalisierungsbezogenen Fortbildungen der Länder ist größer als die in der Studie berücksichtigte Menge. Unsere Suche umschließt Angebote, die sich direkt an Lehrkräfte der Naturwissenschaften richten. Es wurden daher keine Angebote aufgenommen, die allgemeinpädagogischer Natur waren, da damit die fachdidaktische Übertragbarkeit der Fortbildungsangebote erst durch die Lehrkraft gewährleistet werden muss, wodurch die Wahrscheinlichkeit zunimmt, dass die Fortbildungsinhalte nicht auf den beruflichen Alltag übertragen werden (Petko et al. 2018).

Der Zugang zu den Fortbildungsdaten wird von den Bundesländern unterschiedlich gehandhabt, sodass zur Datensammlung verschiedene Wege beschritten werden mussten. Einige Bundesländer (Bayern, Brandenburg, Hamburg, Hessen, Thüringen) stellen ihr Fortbildungsangebot frei zugänglich ins Internet. In den anderen Bundesländern ist diese uneingeschränkte Datenabfrage nicht möglich. In diesen Fällen wurde bei den zuständigen Stellen der Kultusministerien um Zurverfügungstellung des Fortbildungskatalogs gebeten. Zwischen Juli und November 2020 übermittelten Baden-Württemberg, Berlin, Bremen, Mecklenburg-Vorpommern, Rheinland-Pfalz, Saarland und Sachsen-Anhalt die angefragten Daten. Vier Bundesländer stellten uns im Erhebungszeitraum keine Daten zur Verfügung und konnten daher nicht in die Analyse einbezogen werden.

Zunächst wurde geprüft, mit welchen Suchstichworten („computergestützt“, „online“, „digital“) die meisten relevanten Treffer erzielt werden konnten, da die Bezeichnungen für digitalisierungsbezogene Angebote unter den Bundesländern variieren. Es zeigte sich, dass mit dem Stichwort „digital“ die größte und zugleich treffendste Anzahl an Angeboten erfasst werden konnte, wohingegen sich das Stichwort „online“ eher auf das Veranstaltungsformat bezog (z. B. „Lehrerfortbildung online“) und das Stichwort „computergestützt“ eher auf die Fächer Informatik und Technik begrenzt ist. Im Anschluss daran wurden die Ergebnisse nach den Schulfächern Biologie, Chemie, Physik, Naturwissenschaften sowie länderspezifischer Varianten, wie beispielsweise Natur und Technik, selektiert. Tab. 1 erläutert die Suchstrategie exemplarisch am Fortbildungskatalog des Bundeslands Bayern *Fortbildung in bayerischen Schulen* (FIBS).

**Table 1:** 

Ablauf	Beschreibung	Link
1. Aufruf der Homepage	Internetpräsenz des FIBS (Fortbildung in bayerischen Schulen) wurde aufgerufen	https://fibs.alp.dillingen.de
2. Kombinierte Suche: Wahl der Filtereinstellungen	1. Stichworte: digital	https://fibs.alp.dillingen.de/suche/kombiniert.html
	2. Bedingungen: „nur eines der Stichworte muss enthalten sein“	
	3. Ab Monat: Februar 2019	
	4. Anzeige: alle Veranstaltungen	
	5. Pro Seite: alle	
	6. Anbieter: nur staatliche	
	7. Dauer: alle	
	8. Verwendung der Filteroptionen „Fächer“: „Natur und Technik“ „Chemie“ „Physik“ „Umweltbildung“ „Biologie“ „Physik/Chemie/Biologie“	
3. Veranstaltungen aufrufen	Mit der Filteroption „Natur und Technik“ (exemplarisch) werden 10 Veranstaltungen angezeigt	https://fibs.alp.dillingen.de/suche/suche_filter.php
4. Veranstaltungen nach Ausschluss	Die Detailinformationen der angezeigten Veranstaltungen wurden geöffnet. Eingeschlossen wurden Fortbildungsangebote, welche die verbleibenden Kriterien (Zielgruppe, Schulart) erfüllten	https://fibs.alp.dillingen.de/suche/details.php

Von den so identifizierten Fortbildungen wurden, sofern vorhanden, folgende Daten erhoben: Veranstaltungstitel, Zeitumfang, Schulart, Beschreibungstext sowie Format (Online/Präsenz), im Falle von Präsenz der Standort und die verfügbare Teilnehmerzahl.

### Datenauswertung

Die Daten wurden anschließend den Bereichen digitaler Basiskompetenzen des DiKoLAN-Orientierungsrahmens zugeordnet, soweit die Angaben zu den Veranstaltungen hinreichende Informationen enthielten. Hierfür wurde auf der Grundlage von DiKoLAN ein Handbuch zur Kategorisierung entwickelt bestehend aus Kategorien, Definitionen und Beschreibungen der Kategorien sowie aussagekräftigen Beispielen. Alle Fortbildungsdaten wurden anschließend in mehreren Durchgängen von zwei Personen kategorisiert. Bei abweichenden Zuordnungen erfolgte eine konsensuelle Validierung der Daten. Anschließend an die qualitative Auswertung wurden die Daten quantifiziert und deskriptiv analysiert.

Die gleiche Veranstaltung konnte in Abhängigkeit ihrer Zielstellungen mehreren Kompetenzbereichen zugeordnet werden. Es wurde keine Einordnung in die Kompetenzniveaus von DiKoLAN vorgenommen (*Nennen, Beschreiben* sowie *Anwenden/Durchführen*), da die über die Fortbildungsangebote bereitgestellten Informationen eine derart feine Auflösung nicht zuließen.

Zusätzlich wurden anhand der Beschreibungstexte sofern möglich ein fachwissenschaftlicher Schwerpunkt sowie die verwendete Hard- und Software erfasst. Inwieweit eine Fortbildung die Ausbringung von Lernangeboten im Distanzunterricht unterstützt, wurde auf der Basis aller bereitgestellter Daten bewertet. Als *geeignet* eingestuft wurden Fortbildungsangebote, die den Lehrkräften fachliche (CK), (fach-)didaktische (TPK, TPACK) (z. B. digitale Visualisierungsmöglichkeiten im Chemieunterricht) oder technische (TK) (z. B. Nutzung von Cloud-Diensten im naturwissenschaftlichen Unterricht) Handlungsoptionen für den Distanzunterricht ermöglichten. Als *nicht geeignet* eingeordnet wurden Angebote, die die Präsenz im Klassenzimmer erfordern (z. B. Arduino als Steuerzentrale im Physikunterricht).

## Darstellung der Ergebnisse

**Figure 1:**
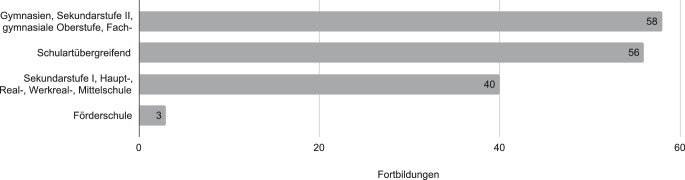

Insgesamt wurden im Zeitraum vom 01.02.2019 bis zum 29.02.2020 aus den Daten von 12 Bundesländern 90 Fortbildungen identifiziert, die einen Bezug zu einem oder mehreren der naturwissenschaftlichen Fächer aufwiesen. 85 Veranstaltungen (94,4 %) wurden in Präsenz abgehalten, fünf online angeboten. Bei der Ermittlung der von den Fortbildungen adressierten Schultypen wurden Mehrfachzuordnungen berücksichtigt (siehe Abb. 1). Knapp 36,9 % der Fortbildungen richteten sich an die Sekundarstufe II. Etwa 35,7 % sind schulartübergreifend und richteten sich beispielsweise an Gemeinschafts‑, Stadteil- oder Integrierte Sekundarschulen. Rund ein Viertel (25,5 %) der Fortbildungsangebote adressierte Schulen der Sekundarstufe I, rund zwei Prozent (1,9 %) war für Förderschulen gedacht.

### Adressierte Kompetenzbereiche

**Figure 2:**
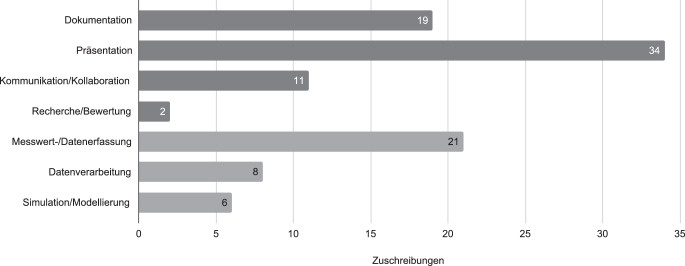

Zur Feststellung der adressierten Kompetenzbereiche wurden die Fortbildungsbeschreibungen anhand der Beschreibungen der DiKoLAN-Kompetenzbereiche analysiert. Eine Zuschreibung wurde vorgenommen, wenn die in DiKoLAN beschriebene Fähigkeit im Beschreibungstext einer Fortbildung identifiziert werden konnte. Durch Nennung mehrere Kompetenzen war es möglich, dass eine Veranstaltung mehreren DiKoLAN-Kompetenzbereichen zugeordnet werden konnte. In der Summe ließen die Zielstellungen der Fortbildungsangebote 101 Zuordnungen zu Kompetenzbereichen des DiKoLAN-Orientierungsrahmens zu (siehe Abb. 2). Ein Großteil der Veranstaltungen adressierte die allgemeineren Kompetenzen *Dokumentation* (19), *Präsentation* (34) sowie *Kommunikation und Kollaboration* (11) mit einem prozentualen Anteil von 65,3 %. Die übrigen 34,7 % entfielen auf die fachspezifischeren Kompetenzen *Messwert- und Datenerfassung* (21), *Datenverarbeitung* (8) und *Simulation und Modellierung* (6).

24 Veranstaltungen konnten aufgrund fehlender Beschreibungstexte keinem Kompetenzbereich zugeordnet werden. Die Beschreibungstexte von drei weiteren Veranstaltungen adressierten wie das folgende Beispiel eher technologisches Wissen, das in den DiKoLAN-Kompetenzbereichen nicht abgebildet ist:

LEGO Education SPIKE Prime ist ein neues digitales Lernkonzept für den Einsatz in naturwissenschaftlich-technischen Fächern und der digitalen Medienbildung. Im Workshop lernen die Teilnehmer die Hardwarekomponenten kennen und bauen selbständig ein einfaches Robotermodell. Sie lösen spannende Problemstellungen aus den originalen Schülermaterialien durch Programmieren der Roboter mit der grafischen Programmiersprache Scratch. Der Einsatz des Systems in Fächern und Klassen wird aufgezeigt. (Fortbildungskatalog Berlin)

### Didaktische Funktionen der Fortbildungsangebote

Die Einordnung der didaktischen Funktionen der Fortbildungsangebote und damit die Zuordnung zu möglichen Lehr-Lern-Szenarien erfolgte anhand der jeweils genutzten Software, inklusive Lernmanagementsysteme (z. B. mebis). Didaktische Funktionen finden im Unterricht Anwendung und werden unterschieden in Lernwerkzeuge, Lernbegleiter, Experimentalwerkzeuge und Lerngegenstände (Huwer et al. 2020). Lernwerkzeuge adressieren primär zeitlich begrenzt einzusetzende Tools, die den Lernprozess fördern sollen (z. B. Erklärvideos). Lernbegleiter dienen der Unterstützung des Lernens, indem sie das Lernen begleiten, ggf. Orte verbinden und in der Regel das Lernen strukturieren (z. B. MOOCs, oder digitale Schulbücher). Experimentalwerkzeuge dienen dazu, den Experimentalprozess im Naturwissenschaftsunterricht direkt anzureichern. Als Lerngegenstand stehen die Medien selbst im Fokus des Lernprozesses. Die didaktischen Funktionen Lernwerkzeuge, Lernbegleiter und Experimentalwerkzeuge adressieren das Lernen mit Medien, während Lerngegenstände das Lernen über Medien adressiert. Diese Funktionen wurden um eine fünfte Funktion – die *Unterrichtsorganisation* – erweitert (siehe Tab. 2). *Unterrichtsorganisation *soll alle Aspekte abseits des eigentlichen Unterrichts erfassen (z. B. der digitalen Verwaltung der Chemikaliensammlung oder Gefährdungsbeurteilungen), die wie die Erstellung von Gefährdungsbeurteilungen in der Vorbereitung einer Unterrichtsstunde liegen und mit denen Schüler*innen nur indirekt oder gar nicht in Kontakt kommen.

**Table 2:** 

	Lernwerkzeug	Lernbegleiter	Experimentalwerkzeug	Lerngegenstand	Unterrichtsorganisation
Cloud	–	★	–	–	★
DEGINTU	–	–	–	–	★
Chemsketch	★	–	–	–	–
H5P	★	★	–	–	–
LearningApps	★	★	★	–	–
Mebis II	–	★	–	–	–
Tet.folio	–	★	–	–	–
Digital.learning.lab	★	★	★	★	★
PhyPiDAQ-Software	–	–	★	–	–
picoDAQ-Software	–	★	★	–	–
PHYWE measureAPP	–	–	★	–	★
Excel	★	–	★	–	–
Digitaler EscapeRoom	–	★	–	–	–
Quizformat Kahoot	★	–	–	–	–
QR-Codes	★	★	–	–	–
Bestimmungshilfen	★	★	–	–	–
Bild‑/Textbearbeitungsprogramme	★	–	–	–	–
Multitouch Learning Books	–	★	–	–	–
Social Media	–	–	–	★	–

### Eignung zur Vorbereitung auf Distanzunterricht

**Figure 3:**
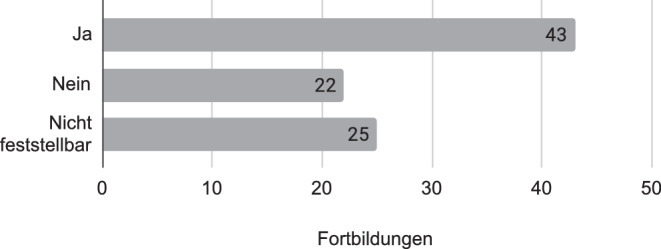

Die Inhalte von 43 der Fortbildungsangebote trugen potenziell dazu bei, Kompetenzen zu schulen, die notwendig oder hilfreich für die Durchführung von Distanzunterricht sind, indem sie Lehrende beispielsweise mit geeigneten digitalen Materialien ausstatteten. 22 Fortbildungen waren an Hard- oder Softwarevoraussetzungen der Schulen gebunden. 25 ließen aufgrund fehlender Beschreibungsinformationen keine Zuordnung zu (siehe Abb. 3).

### Fachliche Schwerpunkte der Fortbildungsveranstaltungen

Lediglich 28 (31,1 %) Fortbildungsangeboten konnten fachliche Schwerpunkte entnommen werden. Diese sind in Tab. 3 gelistet.

**Table 3:** 

Fach	Fachliche Schwerpunkte
Biologie	Genetik Ökologie und Biodiversität (Insektivoren und ihre Bodenbedingungen/Fangmethoden, Artenvielfalt der Lebensgemeinschaften Wiese und Rasen, Bestäubung und Blütenökologie, Klimaschutz, Ökologie und Nachhaltigkeit, Lebensräume und Ökologie, Artenkenntnis und Bestimmen, Regenwurmhaus, Winter-Gartenvögel) Sinnes- und Neurophysiologie (Auge und Nervensystem des Menschen, Neurophysiologie) Zytologie
Chemie	Elektronendichteoberflächen/Donator-Akzeptor-Konzept, Redoxreaktionen, Säure-Base-Reaktionen, Organische Chemie, Elektrochemie, Elektronendichteoberflächen/Donator-Akzeptor-Konzept, Metallbindung und zwischenmolekulare Wechselwirkungen, Moleküle, Kohlenwasserstoffe, Alkohole und Essigsäure, Säuren und Basen, Gleichgewichte in aquatischen Systemen, Atmosphäre und Technik
Physik	Strom, Spannung, Beschleunigung, Druck, Kraft, kosmische Myonen
Naturwissenschaften übergreifend	Boden, Biologische Vielfalt und ökologische Zusammenhänge, Sustainable Development Goals, Bau eines Fahrzeuges, Halbzeug, MINT-Berufsorientierung, Nitrat, Silikat, Ammonium, D‑Glucose, Phytoplankton, NOx, Astronomie

### In Fortbildungen genutzte Hardware

In den Fortbildungen wird mit Notebooks, Tablets und Smartphones gearbeitet. Zur Messwerterfassung wird auf verschiedenste Sensoren und Versuchssysteme unterschiedlicher Hersteller zurückgegriffen (z. B. Leybold Mobile CASSY, PASCO, Phywe, Vernier). Des Weiteren werden Kleinstcomputer (Arduino, Calliope mini), 3D-Drucker oder Lernsysteme (z. B. LEGO Education) thematisiert.

## Diskussion

Ziel der Studie war eine Sichtung und Analyse der für Lehrkräfte der Naturwissenschaften verfügbaren Fortbildungsangebote im Zeitraum von einem Jahr vor den Covid-19 bedingten Schulschließungen im März 2020. Auf der Basis der Angaben aus 12 von 16 Bundesländern konnten 90 Angebote identifiziert werden, die explizit Lehrkräfte der Naturwissenschaften adressieren.

Die betrachteten Bundesländer kommen ihrer Aufgabe nach, Fortbildungsangebote zur digitalisierungsbezogenen Qualifizierung von Lehrkräften auszubringen. Aufgrund der insgesamt niedrigen Zahl an Angeboten ist davon auszugehen, dass nicht alle Naturwissenschaftslehrkräfte in den Bundesländern hinreichend Zugang zu Fortbildungsangeboten erhalten, insbesondere vor dem Hintergrund der vergleichsweise geringen Vorerfahrungen der derzeit im Schuldienst befindlichen Lehrkräfte infolge fehlender Lerngelegenheiten während der ersten und zweiten Phase der Lehrerbildung. Eine kumulative Qualifizierung mit dem Anspruch einer Vertiefung verfügbarer vorhandener Kompetenzen – wie im Strategiepapier „Bildung in der digitalen Welt“ (KMK 2016) vorgesehen – erscheint mit dem beschriebenen Angebot nur eingeschränkt möglich. Die niedrige Anzahl an Fortbildungen lässt zudem vermuten, dass die im Vergleich zu anderen der an den ICILS-Studien beteiligten Länder geringe Teilnahme deutscher Lehrkräfte an Fortbildungsveranstaltungen auch mit dem begrenzten Angebot zusammenhängen könnte. Dem zunehmenden Wunsch von Lehrkräften nach Fortbildungen zu digitalisierungsbezogenen Kompetenzen (Eickelmann et al. 2016; Huber et al. 2020) wird folglich durch das verfügbare Angebot nur zum Teil entsprochen. Zur Erreichung der Zielsetzungen der KMK-Strategie „Bildung in der digitalen Welt“ und damit einer Nachhaltigkeit der eingeleiteten Maßnahmen ist eine Ausweitung des Veranstaltungsangebots unumgänglich.

Der weitaus überwiegende Anteil der Fortbildungen wird in Präsenz ausgebracht (94,4 %), die Anzahl potenziell Teilnehmender ist damit begrenzt. Auch dem aktuell dringenden Bedarf an virtuellen Schulungen wird damit nicht entsprochen. Genaue Angaben zu verfügbaren Plätzen konnten den Daten nicht entnommen werden. Um bei begrenzten finanziellen und personellen Ressourcen eine Angebotsvervielfältigung zu ermöglichen, könnte darüber nachgedacht werden, die sich zum Teil inhaltlich überschneidenden Angebote der Bundesländer stärker als bisher über die Ländergrenzen hinweg zu öffnen und diese auch für die Lehrkräfte aus anderen Bundesländern sichtbar zu machen, etwa indem diese in die jeweilige Fortbildungskataloge aufgenommen werden oder durch Schaffung einer eigenen bundesweiten Professionalisierungsplattform. Unter den erfassten Fortbildungsangeboten waren nur zwei für alle Bundesländern angeboten. Allerdings waren diese beiden Angebote nicht in allen Katalogen sichtbar. Parallel zu einer Öffnung der Angebote könnte, wo immer dies inhaltlich sinnvoll erscheint, über alternative Ausbringungsformen, zum Beispiel über webbasierte Formate, nachgedacht werden, die eine leichtere Vereinbarkeit von schulischer Arbeit und Fortbildung ermöglichen könnten (Eickelmann et al. 2019c) und gleichzeitig das Potenzial hätte, als Vorbildfunktion, wie digitalisierte Lehre aussehen könnte, zu dienen.

### Adressierte Kompetenzbereiche

Bis auf wenige Ausnahmen (*n* = 3) adressieren alle Angebote Kompetenzen aus dem DiKoLAN-Orientierungsrahmen und haben damit das Potenzial, zu einer domänenspezifischen Qualifizierung naturwissenschaftlicher Lehrkräfte beizutragen. Etwa zwei Drittel der Fortbildungen zielte auf die Vermittlung von Kompetenzen in den eher allgemeineren DiKoLAN-Kompetenzbereichen Dokumentation, Präsentation, Kommunikation und Kollaboration sowie Recherche und Bewertung. Unter diesen richten sich 80 % der Angebote an die Bereiche Dokumentation und Präsentation, während die für kognitive Aktivierung, Peer-Feedback, Experten-Feedback und formatives Assessment der Lernenden besonders relevanten Bereiche Kommunikation und Kollaboration sowie Recherche und Bewertung weitaus seltener in Fortbildungen aufgegriffen werden. Dass hier allerdings erhöhter Bedarf ist, lassen die erwähnten Studien von Rannastu-Avalos und Siiman (2020), sowie Eichhorn et al. (2020) vermuten. Die Fortbildungsschwerpunkte unterstreichen sowohl Ergebnisse der ICILS-Studien (Eickelmann et al. 2019a) wie erste Ergebnisse aus dem Lockdown (König et al. 2020), die gerade in diesen Bereichen Lücken in der Nutzung digitaler Technologien sehen, die durch darauf bezogene Qualifizierungsangebote adressiert werden sollten.

Digitalisierungsbezogene Fortbildungen zu eher fachspezifischeren Kompetenzbereichen werden zwar angeboten, doch ist ihre Anzahl im Vergleich zu den eher allgemeineren Fortbildungen deutlich geringer (35,7 %). Noch etwas geringer (31,1 %) ist die Zahl der Fortbildungen, die fachliche Schwerpunkte von Veranstaltungen benennt und damit Lösungen für domänenspezifische Herausforderungen expliziert, für die digitale Medien genutzt werden können.

Die Fortbildungsangebote richten sich in erster Linie an die Nutzung digitaler Medien zur Verbesserung von Lehr-Lern-Prozessen. Es kann sein, dass in den Fortbildungen auch andere Zielperspektiven des Einsatzes digitaler Medien wie etwa die Entwicklung und Umsetzung neuer Formen des Unterrichtens (Eickelmann und Gerick 2020) aufgegriffen werden, jedoch sind diesbezügliche Informationen den Angebotsbeschreibungen nicht zu entnehmen.

Zumindest den in diese Studie eingegangen Fortbildungsangeboten lässt sich kein Rückschluss auf einen Kompetenzrahmen des jeweiligen Bundeslandes entnehmen, der interessierten Lehrkräften ein individuelles (Selbst‑)Assessment ihrer Kompetenzen und in der Folge einen kumulativen Kompetenzaufbau über eine individuelle Fortbildungsplanung ermöglichen könnte. Ohne eine solche Orientierung erscheint es aber eine herausfordernde Aufgabe für Lehrkräfte, die für sie relevanten Kompetenzen zu identifizieren und ihre (Weiter‑)Qualifikation zielgerichtet zu steuern. Vor diesem Hintergrund könnte es hilfreich sein, wenn die Bundesländer ihre zum Teil bereits seit einiger Zeit definierten Kompetenzrahmen für Lehrkräfte noch stärker sichtbar machen und als Orientierungsrahmen anbieten (z. B. Forschungsgruppe Lehrerbildung Digitaler Campus Bayern 2017).

Eine Herausforderung für diese Studie war die Nutzung der Beschreibungen zu den Fortbildungen zur Identifikation von Inhalten und zu erwerbenden Kompetenzen. So weist mehr als ein Viertel der erfassten Fortbildungsveranstaltungen (26,7 %) überhaupt keine inhaltlichen Beschreibungen auf. Damit Lehrkräfte die von der Fortbildung adressierten Inhalte und Kompetenzen überhaupt identifizieren und in ein Kompetenzmodell einordnen zu können, erscheint es notwendig, den Fortbildungsangeboten in dieser Hinsicht aussagekräftige Beschreibungen hinzuzufügen. Möglicherweise könnte bereits dadurch der Eindruck von Lehrkräften abgemildert werden, dass für sie keine relevanten digitalisierungsbezogenen Angebote existieren (GEW 2020).

### Vorbereitung Distanzunterricht

Abschließend bleibt die Frage, inwiefern die erfassten Lehrerfortbildungen auf den Distanzunterricht vorbereitet haben. Hierzu wurden die didaktischen Funktionen der Fortbildungen betrachtet. Für eine solche Bewertung ist zudem zu berücksichtigen, dass keine der Fortbildungen mit dem Ziel angeboten werden konnte, die Lehrkräfte auf eine pandemiebedingte Schulschließung vorzubereiten. Dennoch konnten bei der Analyse zentrale Aspekte identifiziert werden, die beim Distanzunterricht helfen konnten, indem sie digitale Medien als Lernbegleiter vorstellten, wozu beispielweise die Nutzung von Lernplattformen zählt. Immerhin hatten 43 der analysierten Lernangebote (und damit 47,7 %) das Potenzial, auf den Distanzunterricht vorzubereiten. Es besteht also eine Grundlage, auf der sich aufzubauen lohnt.

## Limitationen und Herausforderungen

In die Studie eingegangen sind die in staatlichen Fortbildungskatalogen aufgeführten digitalisierungsbezogenen Fortbildungen in den drei Naturwissenschaften.

## Fazit

Die in die Untersuchung einbezogenen Bundesländer bringen Fortbildungsangebote zur Vermittlung von digitalisierungsbezogenen Kompetenzen für ihre Lehrkräfte aus. Allerdings zeigt sich Entwicklungsbedarf hinsichtlich der Anzahl der Angebote, ihrer Orientierung an Kompetenzrahmen und wo vorhanden der Sichtbarkeit der Kompetenzrahmen, die Lehrkräften eine kumulative Kompetenzentwicklung erleichtern könnte, sowie eine klare Differenzierung zwischen fachspezifischen und fachunspezifischen Angeboten. Die Angebote selbst leiden häufig unter fehlenden oder wenig aussagekräftigen Beschreibungen und sie werden von ganz wenigen Ausnahmen abgesehen länderspezifisch ausgebracht, auch wenn sie länderübergreifende Relevanz haben. Die bundesweit verfügbaren Angebote sind nicht in allen Fortbildungskatalogen der Länder aufgeführt und damit für einen Teil der Lehrkräfte im Wortsinne unsichtbar. Für die rasche Erreichung der Zielsetzungen der KMK-Strategie „Bildung in der digitalen Welt“ sind mehrere Empfehlungen denkbar: 1. Rasche Erhöhung der Anzahl der verfügbaren Fortbildungen, wo möglich, z. B. für die Erläuterung von digitalen Tools, über webbasierte Fortbildungen, 2. Schaffung und Sichtbarmachung einer bundesweiten Fortbildungsplattform für digitalisierungsbezogene Angebote, 3. Festlegung auf einen Kompetenzrahmen zur digitalisierungsbezogenen Qualifikation von Lehrkräften und Ausrichtung des Fortbildungsangebots an einem solchen Rahmen, 4. Ergänzung der bundesweiten digitalen Angebote um transferbezogene regionale Angebote, die die Kompetenzentwicklung von Lehrkräften im Unterricht entsprechend der Schulsituation vor Ort unterstützen.
